# Comprehensive Evaluation of the Anti-Angiogenic and Anti-Neoplastic Effects of Endostar on Liver Cancer through Optical Molecular Imaging

**DOI:** 10.1371/journal.pone.0085559

**Published:** 2014-01-08

**Authors:** Qian Zhang, Yang Du, Zhenwen Xue, Chongwei Chi, Xiaohua Jia, Jie Tian

**Affiliations:** 1 School of Life Sciences and Technology, Xidian University, Xi’an, Shaanxi, China; 2 Institute of Automation, Chinese Academy of Sciences, Beijing, China; Stanford University, United States of America

## Abstract

Molecular imaging enables non-invasive monitoring of tumor growth, progression, and drug treatment response, and it has become an important tool to promote biological studies in recent years. In this study, we comprehensively evaluated the *in vivo* anti-angiogenic and anti-neoplastic effects of Endostar on liver cancer based on the optical molecular imaging systems including micro-computer tomography (Micro-CT), bioluminescence molecular imaging (BLI) and fluorescence molecular tomography (FMT). Firefly luciferase (fLuc) and green fluorescent protein (GFP) dual labeled human hepatocellular carcinoma cells (HCC-LM3-fLuc-GFP cells) were used to establish the subcutaneous and orthotopic liver tumor model. After the tumor cells were implanted 14∼18 days, Endostar (5 mg/kg/day) was administered through an intravenous tail vein injection for continuous 14 days. The computer tomography angiography (CTA) and BLI were carried out for the subcutaneous tumor model. FMT was executed for the orthotopic tumor model. The CTA data showed that tumor vessel formation and the peritumoral vasculature of subcutaneous tumor in the Endostar treatment group was significantly inhibited compared to the control group. The BLI data exhibited the obvious tumor inhibition day 8 post-treatment. The FMT detected the tumor suppression effects of Endostar as early as day 4 post-treatment and measured the tumor location. The above data confirmed the effects of Endostar on anti-angiogenesis and tumor suppression on liver cancer. Our system combined CTA, BLI, and FMT to offer more comprehensive information about the effects of Endostar on the suppression of vessel and tumor formation. Optical molecular imaging system enabled the non-invasive and reliable assessment of anti-tumor drug efficacy on liver cancer.

## Introduction

Hepatocellular carcinoma (HCC) is one of the most common cancer in the world and is responsible for more than 600,000 deaths annually [1]. Unfortunately, the disease is often detected at a late stage, when potentially curative therapies are often ineffective [2]. Most cancer patients show disease recurrence that rapidly progresses to the advanced stages with vascular invasion and their 5-year relative survival rate is only 7% [3]. Therefore new therapies and new detection methods for this aggressive disease are extremely needed.

Angiogenesis is required for invasive tumor growth and metastasis, so it plays an important role in the control of cancer progression [4], [5]. The rapid growth of the tumor needs a large amount of nutrients and oxygen, which prompted the growth of blood vessels. Moreover, HCC tumors depend on a rich blood supply [6], [7], therefore, inhibition of angiogenesis has constituted a crucial point in liver cancer therapy.

Preclinical studies have shown that endostatin could shrink existing tumor blood vessels effectively [4], [8]–[10]. Endostar is a novel recombinant human endostain expressed and purified in *Escherichia coli* with a modified N-terminal [11], [12], it has been shown to inhibit endothelial cell proliferation, migration, and vessel formation [13]. Based on systemic preclinical studies and clinical trials, the State Food and Drug Administration of China (SFDA) approved the regimen for the treatment of non-small-cell lung cancer in 2005 [14]. In this study, we explored the antitumor effects of Endostar on liver cancer *in vivo*.

The traditional approach for anti-neoplastic research such as histopathological analysis is accurate but time-consuming and cannot provide 3-dimensional (3D) structural information. Furthermore, some anti-angiogenic agents usually inhibit tumor progression rather than tumor volume shrinkage. Therefore, the evaluation of therapeutic response only through tumor volume measurement is no longer comprehensive [15], and it is an urgent need to develop a more sensitive and effective detection method.

Molecular imaging, which allows for sensitive, longitudinal observation, has become an invaluable tool for early lesion detection, monitoring therapeutic efficacy, and facilitating drug development. Noninvasive imaging of tumor angiogenesis allows for much earlier diagnosis and better prognosis, which will eventually lead to effective therapy [16]. In recent years, optical molecular imaging has emerged as an important tool of technologies to advance our understanding of disease mechanisms and accelerate drug discovery [17], [18]. We combined CTA, BLI and FMT to overcome the imaging depths and resolution limits, which allowed researchers to pinpoint the tumor progression and location [19], [20].

Since it is the suitable way to represent the disease process in human, orthotopic animal models of human cancer are the optimal tool for preclinical evaluation of novel therapies [21]. Although the orthotopic liver cancer model has been studied extensively [22], [23], its application in optical molecular imaging for longitudinal monitoring and drug response on liver cancer has seldom been tried. Through our Micro-CT/BLI/FMT system, CTA was carried out to analyze vascular 3D morphological changes and give the quantitative assessment. Due to the minimal background of BLI, it was used for the imaging of subcutaneous tumor model. Taking advantages of transillumination mode and sophisticated computer algorithms, the FMT is to reconstruct 3D maps of fluorophores inside living animals [24]. We chose FMT to provide the tumor location and volume information for the orthotopic tumor model. When the data acquisition was completed, the results were reconstructed through our independently developed imaging software. The optical molecular imaging system permitted facile and early detection of tumor progression and therapeutic responses, and we presented a comprehensive evaluation of the anti-tumor effect of Endostar.

## Materials and Methods

### Materials and Reagent

Endostar was obtained from the corporation of Simcere (Nanjing, China). D-Luciferin was bought from Biotium (CA, Fremont, USA). Fenestra VC was purchased from ART (Advanced Research Technologies Inc., Canada). The dual-labeled human hepatocellular carcinoma cell line HCC-LM3-fLuc-GFP was kindly provided by Prof. Jian Zhao of Shanghai Second Military Medical University [25]. No significant difference was observed between HCC-LM3 and HCC-LM3-fLuc-GFP cells in terms of proliferation, tumorigenicity and migration.

### Cell Culture

HCC-LM3-fLuc-GFP cells were cultured in Dulbecco’s modified eagle medium (DMEM; Thermo Scientific) and supplemented with 10% fetal calf serum (FCS) (HyClone; Thermo Scientific). They were maintained at 37°C incubator with 5% CO_2_.

### Animal Model

4–5 weeks old athymic male BALB/c nude mice (N = 36) were purchased from the Department of Experimental Animals, Peking University Health Science Center. All animal experiments were performed in accordance with the guidelines of the Institutional Animal Care and Use Committee (IACUC) at Peking University (Permit Number: 2011-0039). The research procedures were approved by IACUC of Peking University. All surgery was performed under sodium pentobarbital anesthesia, and all efforts were made to minimize suffering.

The subcutaneous tumor model was established by injecting 1×10^7^ cells/ml HCC-LM3-fLuc-GFP cell suspension 100 ul into the right upper flanks of BALB/c nude mice (N = 24).

The orthotopic tumor model mice (N = 12) was established after the mice had been anesthetized with sodium pentobarbital. A laparotomy was carefully performed and the liver was completely exposed. 1×10^6^ cells/ml HCC-LM3-fLuc-GFP cell suspension 50 ul were injected into the left liver lobe. Post injection bleeding and tumor cell escape were avoided by brief local compression. The abdominal wall and skin were closed with a continuous suture.

### Endostar Treatment

The subcutaneous tumor-bearing mice were randomly divided into the experimental group and control group (N = 12 per group) until tumor volume(volume = 0.5×a×b^2^,where a and b were the respective longer and shorter diameters of the tumor) increased 100 mm^3^ in around 18 days. The orthotopic tumor-bearing mice were randomly divided into the experimental and control group (N = 6 per group) 14 days post-plantation. The experimental group and control group for the subcutaneous and orthotopic tumor-bearing mice were given intravenous tail injections with Endostar (5 mg/kg/day) or an equal amount of 0.9% saline for 14 days. In order to avoid the tumor metastasis, orthotopic tumor group was given Endostar treatment earlier than the subcutaneous tumor group.

### Mouse Body Weight and Tumor Volume

The mouse body weight was measured by the electronic balance every two days. The tumor volume was measured by caliper every four days.

### Histological Studies

Mice were sacrificed at the end of the experiment on day 14 post-treatment. The liver and tumor tissues were excised, fixed in formalin, embedded in paraffin and continuous sections (4 *μ*m) were obtained for hematoxylin and eosin (H&E) staining.

### Immunohistochemistry of CD31

Subcutaneous tumor tissues were excised at the end of the experiment. Specimens were fixed in formalin and embedded in paraffin. 4 *μ*m sections were cut from each block and incubated with 0.01 M natrium citricum for antigen retrieval. The slides were rinsed in phosphate-buffered saline (PBS) and incubated overnight at 4°C with anti-diluted cluster of differentiation 31 (anti-CD31) antibody. The samples were then rinsed twice with phosphate-buffered saline with Tween-20 (PBST) and incubated for 60 min at room temperature with the appropriate dilution of the secondary antibodies. The slides were rinsed with PBST and incubated for 10 min with diaminobenzidine. The sections were then counterstained with hematoxylin and mounted, and pictures were taken under microscope.

### The Tumor Microvessel Density (MVD) Assessment

The slides were examined under the microscope, and 3 fields were randomly taken for microvessel counting and the anti-angiogenic effects of Endostar evaluation.

### Micro-CT/BLI/FMT System

**Figure 1:**
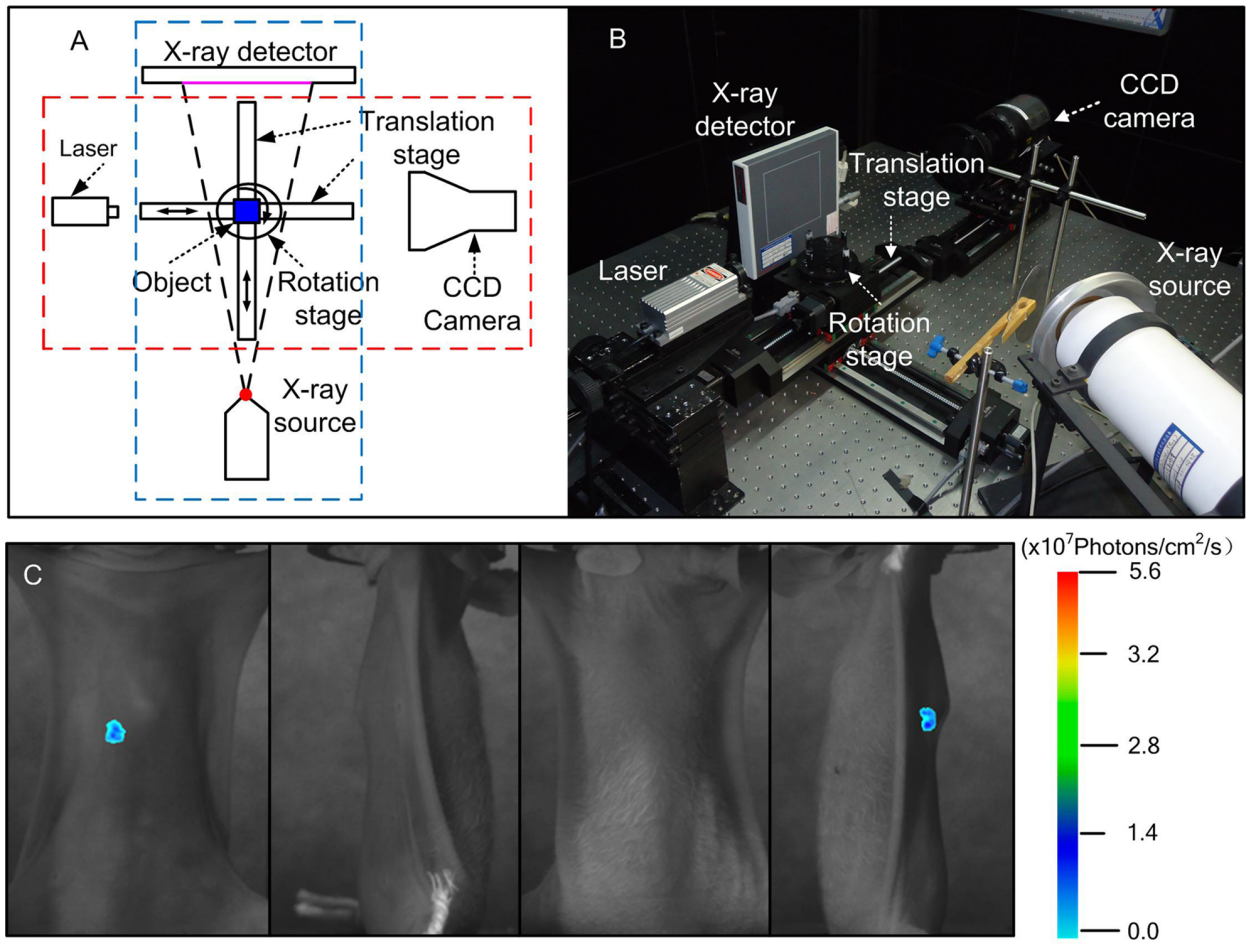
The Micro-CT/BLI/FMT system and the process of FMT imaging. A. The schematic of the multi-modality system. The red dashed line represents the BLI/FMT system; the blue dashed line represents the Micro-CT system. B. The figure of the multi-modality system, which is located in the black lead housing. C. The planar fluorescent imaging of mouse orthotopic tumor model at 0°degree, 90°degree, 180°degree and 270°degree.

Our Micro-CT/BLI/FMT system was shown in Fig. 1A and B. The BLI and FMT system is fusional, and the optical detector is a scientific charge-coupled device (CCD) camera (VersArray, Princeton Instruments, Trenton, New Jersey) with the temperature cooled to −110°C to reduce dark current noise. The CCD camera coupled with a lens (Nikon, Japan). Excitation illumination was provided by a 488 nm continuous-wave (CW) semiconductor laser VA-II-N-473 (Beijing Viasho Technology Co. Ltd, China) and the power was set to 20 mW. The fluorescence measurements were implemented in transillumination mode. A 20 nm band-pass filter centered at 525 nm was used to allow light transmission at the emission wavelength. Without the laser source, the bioluminescence measurements are acquired by this CCD camera directly. The Micro-CT system consists of a micro-focus X-ray source (UltraBright, Oxford Instruments, USA). The target voltage of the X-ray tube is 90 kVp, with a maximum output power of 80 W. The X-ray flat panel detector (C7942CA-02, Hamamatsu, Japan), had a 120 mm×120 mm photodiode area. The 3D programmable stage includes two motorized translation stages (PSA200-11, Zolix Instruments, China) and one motorized rotation stage (RAK-100, Zolix Instruments, China). The magnification ratio can be changed by adjusting the position of the object through the motorized translation stages, which will improve spatial resolution obviously [26]. The home-made mouse rack was fixed on the rotation stage of the multi-modality system. The multi-modality imaging system was located in a dark lead room that can block both external lights and X-rays.

### CTA of Mouse Subcutaneous Tumor Model

CTA was performed on the 14^th^ day when the daily treatment was completed. The mice were anesthetized with 2% isoflorane, and 200 µl vascular contrast agent Fenestra VC was injected into the tail vein. Ten minutes post-injection, 500 projection views were collected by the Micro-CT system. The CT data acquisitions and processing were performed using the Windows Molecular Imaging System (WINMI) [27], which was developed based on the Medical Imaging Tool Kit (MITK) (Medical Image Processing and Analyzing of Science, Beijing, China, www.mitk.net). The CT data gave the segmentation of tumor vessels and reconstructed the 3D structure. Parameters were set as following: the voltage of the X-ray tube was 80 kVp and the detector integration time was 0.467 sec, which captured one image every 0.75°.

### *In vivo* BLI of Mouse Subcutaneous Tumor Model

The BLI was carried out to the subcutaneous mouse models on the 0^th^, 4^th^, 8^th^, and 12^th^ day after daily treatment began. The mice were fasted overnight prior to the experiment to prevent food from interfering with the bioluminescence results. The mouse was first anesthetized with 2% isoflurane and received D-luciferin solution (150 mg/kg body weight) 3 min before BLI was started. The mice were kept in right lateral recumbency. The parameters of CCD were *f*_num = _2.8, binning = 4, exposure time = 180 sec.

### *In vivo* FMT of Mouse Orthotopic Tumor Model

The FMT was implemented to the orthotopic tumor mouse models also on the 0^th^, 4^th^, 8^th^, and 12^th^ day after the daily treatment began. The mouse was affixed on the mouse rack, which was located on the rotation stage of the multi-modality system. 2% isoflorane was given to the mouse through a respiratory mask, which was attached to the mouse rack. The surface measurement of the fluorescent signals was obtained first. The excitation illumination was conducted with a 488 nm continuous wave semiconductor laser with the output power of 20 mW. The acquisition parameters of CCD were set as *f*_num_ = 3.5, binning = 1,exposure time = 10 sec. It was useful for accurate reconstruction of four images (0°degree, 90°degree, 180°degree and 270°degree), which were obtained without being blocked or moved (Fig. 1C). Then, 3D anatomical data was acquired using the Micro-CT system. The parameters were set as following: voltage of the X-ray tube was 60 kVp, the detector integration time was 0.467 sec and one image was captured every 0.75°.

The reconstruction method based on homotopy was used to obtain the results for fluorescence tomography [28]. Due to the adaptive regularization strategy, the reconstruction method was always able to reconstruct sources accurately independent of the estimation of the regularization parameter. As our best knowledge, this method was seldom applied on orthotopic liver tumor model. The main process of this reconstruction algorithm was as follows:

Firstly, the vector of the residual correlations was computed:
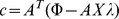



Secondly, the supporting set was updated:
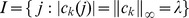



Thirdly, the direction for the next iteration was obtained by:
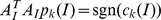



Fourthly, the step for the next iteration was calculated. The minimum were taken only over positive arguments both for the two steps.
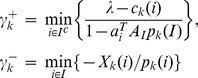



The reconstruction algorithm determines to either append the element to I or remove the element from I depending on which is smaller.
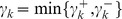



Finally, the unknowns and the regularization parameter were updated.
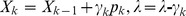



### Statistical Analysis

Data were expressed as mean ± standard deviation (SD). Means were compared using one-way analysis of variance (ANOVA) by using Prism 4.0 software, and *p* value <0.05 were considered statistically significant.

## Results

### Angiogenesis Inhibition was Detected and Monitored by CTA during Endostar Treatment

**Figure 2:**
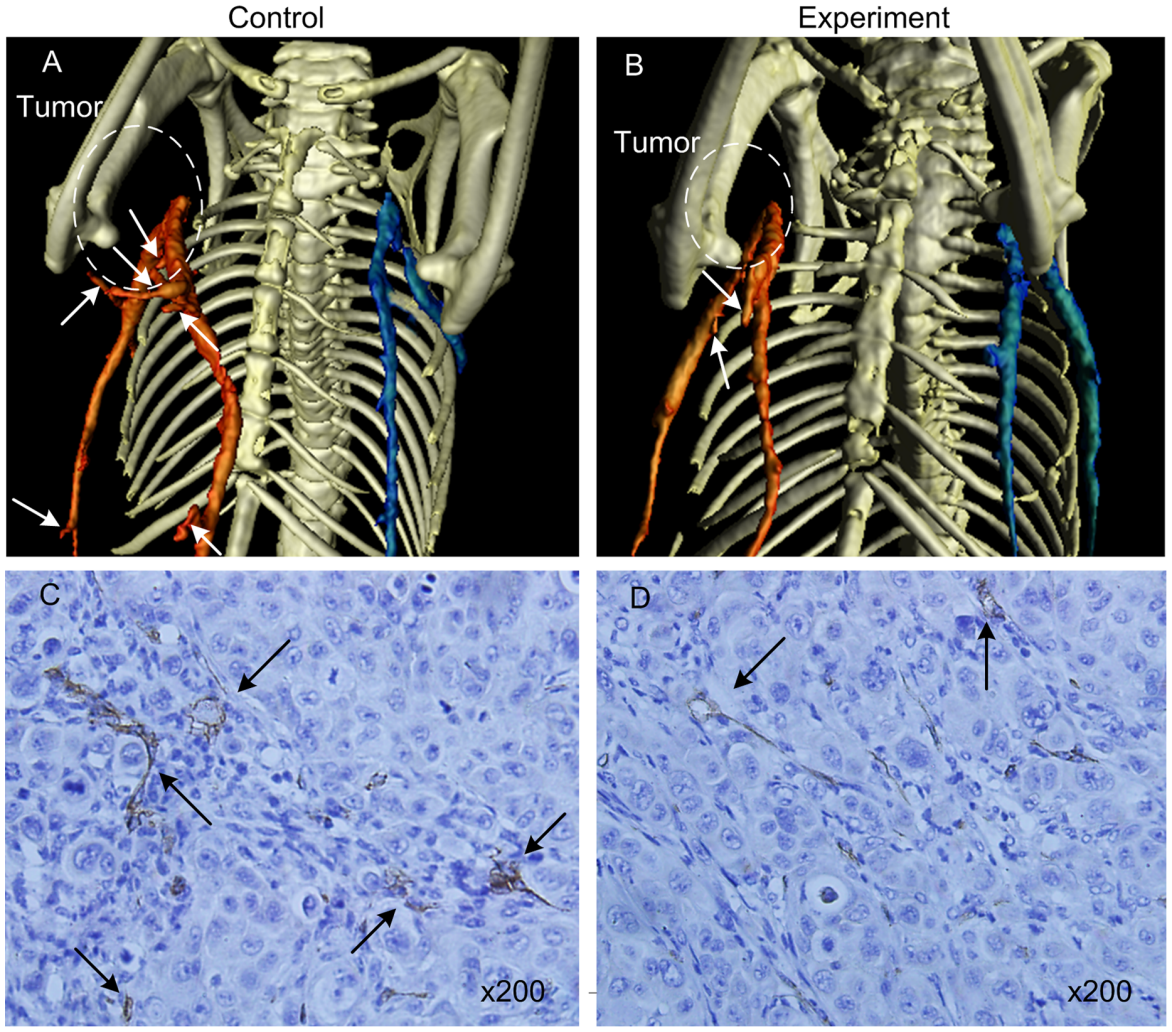
The 3D segmentation images and CD31 immunohistochemistry of the tumor vessels. A and B show the 3D segmentation images by CTA The white arrows show the microvessel sprouting, the blood vessel near the tumor was marked in red, and the blood vessel away from the tumor was marked in blue. C and D show CD31 immunohistochemistry of the tumor vessels. The black arrow indicates the microvascular areas.

With the assistance of the blood pool contrast agent, vascularity was revealed in exquisite detail through CTA. A 3D representation of vascular structures reflected the level of angiogenesis and provided a quantitative assessment of tumor growth over time. We captured the detail of the vasculature change even when the mature tumor vessel was not rich. As shown in Fig. 2A and B, in order to show the different vessels around the tumor, the blood vessel near the tumor was marked in red, and the blood vessel away from the tumor was marked in blue. The microvascule of control group was richer than the experiment group indicated by the white arrow. Although CTA cannot get morphological changes of tumor capillaries as small as scanning electron microscopy (SEM), CTA was a non-invasive method to obtain continuous and dynamic observation of tumor capillaries, which was important for drug evaluation.

Pathological analysis is the gold standard for reflecting tissue lesions. Immunohistochemistry staining of CD31 was done to evaluate the effect of Endostar on tumor angiogenesis. Treatment with 5 mg/kg/day of Endostar resulted in a 34.7% decrease in MVD compared to control group. Representative histological images of CD31 staining on tumor tissues were shown in Fig. 2C and D. The microvasculature of tumor treated with Endostar was smaller in diameter, and CD31 positive cell number was less in the Endostar treatment compared to control. The CD31 immunohistochemistry result was consistent with CTA data, and both of them confirmed that the anti-angiogenic effect of Endostar on liver cancer could be detected and assessed by CTA.

### Neoplastic Inhibition was Monitored by BLI during Endostar Treatment

**Figure 3:**
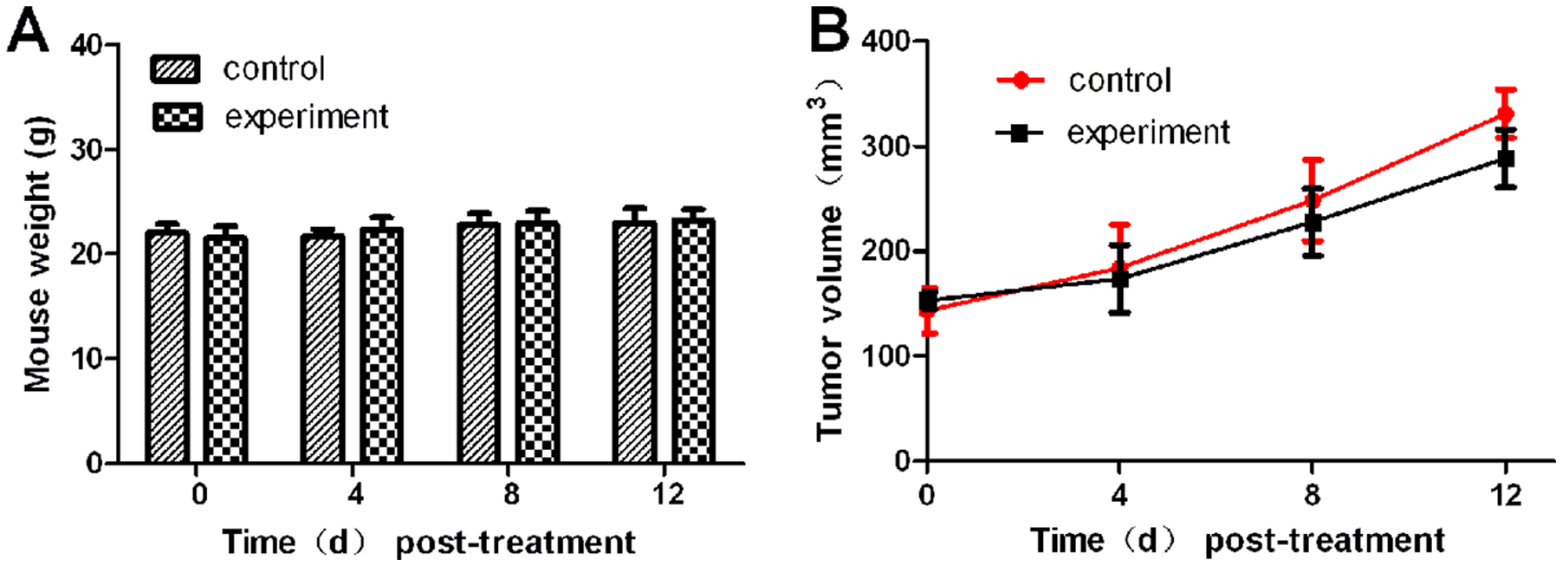
The mouse body weight and tumor volume. A. The mouse body weight of the subcutaneous tumor model. B. The mouse tumor volume of the subcutaneous tumor model.

There was no statistically significant change in body weight between animal groups (Fig. 3A), which indicated that the dosing regimens were well tolerated. The tumor volume was shown in Fig. 3B. In order to track tumor progression and regression more accurate, we not only measured tumor volume, but also performed BLI to record the dynamic light intensity changes.

**Figure 4:**
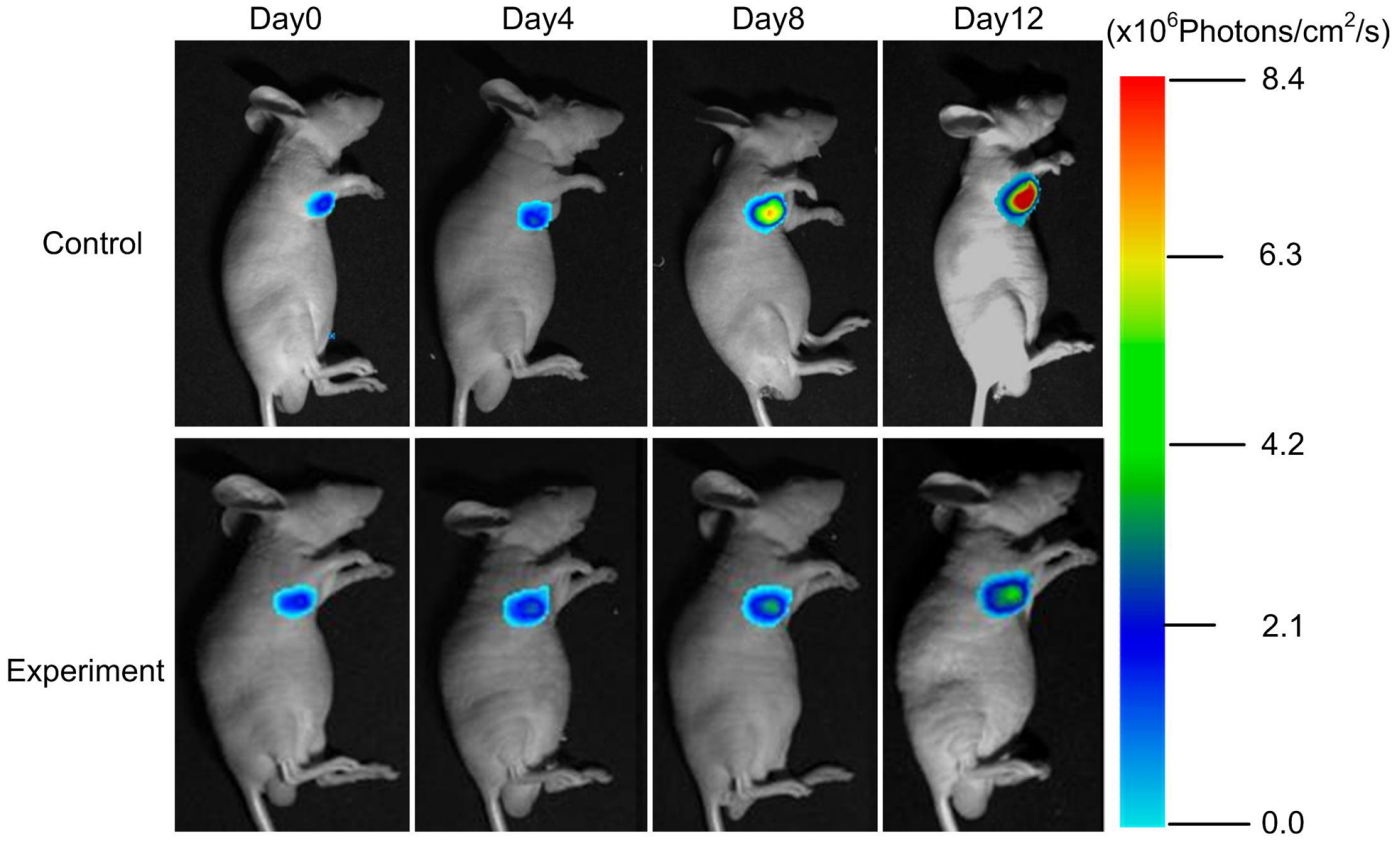
The BLI of subcutaneous tumor model. Continuous BLI light intensity observation of the subcutaneous tumor model from day 0 to day 12.

The results showed that the BLI signal of the experiment group was significantly inhibited since day 8 post-treatment (Fig. 4), while the light intensity of the control group increased exponentially. The most obvious difference between the two groups was obtained on day 12.

**Figure 5:**
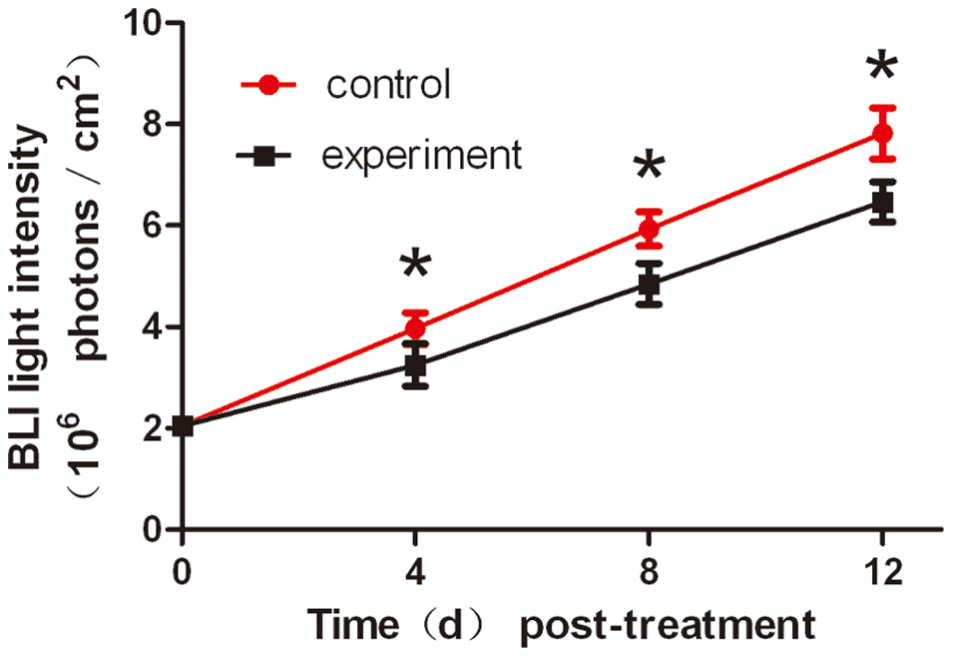
The quantitative BLI light intensity of the subcutaneous tumor.

The subcutaneous tumor BLI light intensity was shown in Fig. 5 The tumor light intensity suppression could be detected as early as day 8 post-treatment (P<0.05). The control group light intensity was 7.8×10^6^ Photons/cm^2^/s on day 12, whereas the experiment group was only 5.6×10^6^ Photons/cm^2^/s.

**Figure 6:**
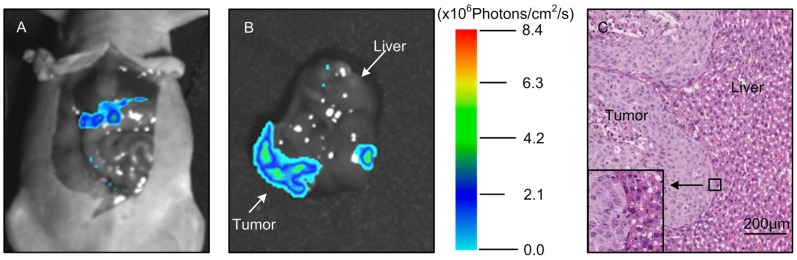
Verifying the validity of the orthotopic tumor model. Laparotomy was performed on the mouse with the liver exposed in the field of view A, and then the liver was dissected. BLI was carried out again and the optical signal was distributed in the hepatic lobular margins in B. C. The H&E stain revealed that there were tumor nodes in the liver tissue.

For the purpose to give a fully anti-tumor evaluation of Endostar, we set up the liver orthotopic tumor model, which preserves the maximum extent of cancer’s “natural” microenvironment [29]–[31]. We anesthetized 2 mice on day 14 post-implantation, and then D-luciferin was injected intraperitoneally 3 min with their liver exposed before BLI (Fig. 6A). There was an intense signal from the hepatic lobular margins. We then excised the liver for BLI immediately, and the tissue remained illuminated (Fig. 6B). The liver tissue was then H&E stained, and it showed that liver tumors located in the hepatic lobes (Fig. 6C). The results proved the orthotopic liver tumor model was successful.

### Monitoring of Neoplastic Inhibition by FMT during Endostar Treatment

**Figure 7:**
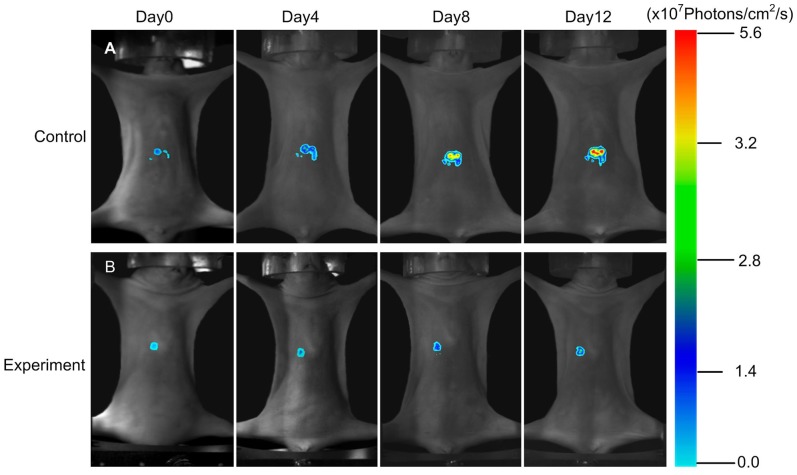
The FMT of the orthotopic tumor model. The coronal degree view of the orthotopic tumor progression and drug response was monitored by the FMT system.

**Figure 8:**
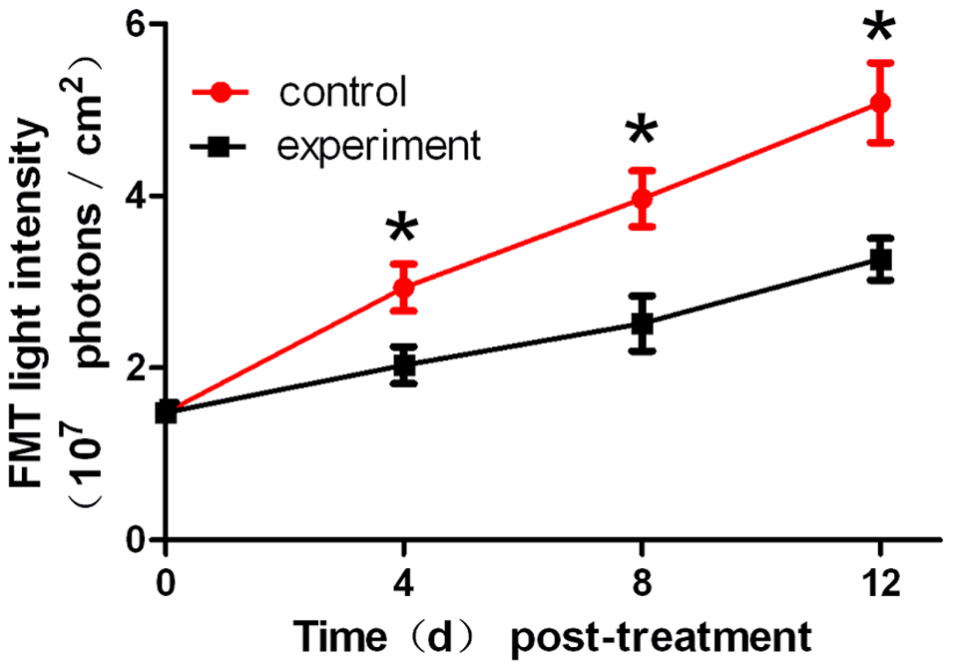
The quantitative FMT light intensity of the orthotopic tumor.

Based on the above results, we further used FMT system to detect the 3D dynamic fluorescent changes in the orthotopic tumor model. Firstly, from coronal degree imaging of the 4 time points,a significant light intensity difference was detected as early as 4 day post-treatment between control and experiment group (Fig. 7A and B). Since day 8, signals from the saline group increased dramatically, but the signals from the experiment group grew slowly. On day 12 (Fig. 8, P<0.05), the FMT light intensity of the experiment group was 3.27×10^7^ Photons/cm^2^/s, whereas the control group was as high as 4.98×10^7^ Photons/cm^2^/s.

**Figure 9:**
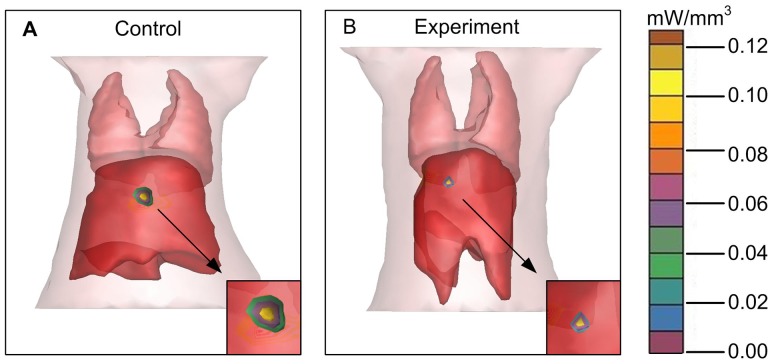
The 3D FMT reconstruction of the orthotopic tumor model. A and B. The FMT reconstruction of the orthotopic tumor model and the partial enlarged view.

For the orthotopic tumor, planar fluorescent imaging was inadequate. Because the weak sources near the surface maybe appear the same surface light intensity to the stronger sources deeper in the tissues [32]. FMT was required for tumor localization and distribution inside a small animal to produce accurate tomographic reconstruction and visualization in 3D mode. We segmented the lung and liver through CT data. The reconstruction results were shown in Fig. 9A and B, where the tumor was located in the liver lobe margin. The power of the experiment group was 2.27 mW, while the control group was 3.83 mW. MITK was used to reconstruct the tumor volume. The control group was 156 mm^3^, while the experiment group was 107 mm^3^. From the above data, it suggested that the FMT provides the accurate location and quantitative analysis of the deep light source, and it served as an ideal method for the deep tissue detection.

## Discussion

Optical molecular imaging has several advantages over traditional methods, especially in the field of *in vivo* cancer research. It can especially enhance the visualization, characterization and quantification of biological processes in living subjects [33]. These features are highly valuable for preclinical tumor research especially for the assessment of new anti-tumor drugs.

Comparing Fig. 3B with Fig. 4 and 5, we found that the differences of the light intensity detected by BLI between control and experiment groups were more dramatic than the tumor volume measured by calipers. The reason was that the bioluminescent light signal emitted from the tumor cells was derived from living tumor cells, which provided a quantitative surrogate measurement of the number of living tumor cells [34], [35]. Therefore, BLI was more sensitive than traditional measurement when the tumor inhibition was not fully reflected in the tumor volume shrinkage. FMT was not only able to reconstruct the tumor location but also to calculate the tumor volume (Fig. 9). Thus for the deep tissue orthotopic tumor, tomography was more suitable.

The roles of CTA, BLI and FMT are complementary to each other, we used the optical molecular imaging system to make a more comprehensive assessment of Endostar on anti-angiogenesis and suppression of hepatoma growth. Firstly, with the help of a blood pool contrast agent and software MITK, the CTA enabled us to obtain a quantitative assessment of vessel structure. This method allows for continuous and non-invasive angiogenesis research. Secondly, the bioluminescent light emitted from tumor cells was derived from metabolically active tumor cells, thus reliable detection such as some anti-angiogenic agents usually inhibit tumor progression rather than tumor volume shrinkage can be achieved by BLI. As a result, BLI is sensitive enough to detect the obvious tumor inhibition of Endostar group as early as day 8. Thirdly, the tumor location can be reconstructed through FMT, which is less depth dependent. For the liver tumor, FMT was able to reconstruct the 3D fluorescence distribution and provide accurate quantification as in previous studies [36], [37]. Therefore the combination of CTA, BLI and FMT enabled us to improve the accuracy of Endostar anti-cancer efficacy.

Our future work direction is to further optimize the resolution of our Micro-CT. The conventional histology of tumor vessels requires sacrificing large number of animals at different time points, then takes time for slicing and staining tissues to identify the location and the density of the vessels. Therefore, if CTA is able to detect miniscule vascular changes, it may become a real-time method to estimate the *in vivo* anti-angiogenesis effect continuously, which is an effective way to reduce costs and save time. At the same time we will improve the reconstruction accuracy to better solve the problem of high absorption of light in deep tissue and integrate different molecular imaging modalities.

## Conclusion

Optical molecular imaging was used from a multi-angle to verify the effects of Endostar in liver cancer inhibition. The integration of a multi-modality system meets the requirements of being noninvasive, accurate, and capable of continuously monitoring Endostar anti-cancer research and drug evaluation. The subcutaneous and orthotopic mouse tumor model simultaneously served as comprehensive tool on the evaluation of Endostar therapeutic research. The combination of functional information with conventional anatomical visualization could accurately characterize lesions, and provides noninvasive biomarkers of therapeutic performance and patient prognosis. The development of further optical molecular imaging facilities our more comprehensive observation of cancer progression and accelerates the discovery of new treatment.

## References

[1] Jemal A, Bray F (2011) Center MM, Ferlay J, Ward E, et al (2011) Global cancer statistics. CA: a cancer journal for clinicians 61: 69–90. 21296855 10.3322/caac.20107

[2] Liu L, Cao Y, Chen C, Zhang X, McNabola A, et al (2006) Sorafenib blocks the RAF/MEK/ERK pathway, inhibits tumor angiogenesis, and induces tumor cell apoptosis in hepatocellular carcinoma model PLC/PRF/5. Cancer Res 66: 11851–11858. 17178882 10.1158/0008-5472.CAN-06-1377

[3] Bosch FX, Ribes J, Diaz M, Cleries R (2004) Primary liver cancer: worldwide incidence and trends. Gastroenterology 127: S5–S16. 15508102 10.1053/j.gastro.2004.09.011

[4] Folkman J (2002) Role of angiogenesis in tumor growth and metastasis. Semin Oncol 29: 15–18. 10.1053/sonc.2002.37263 12516034

[5] Weis SM, Cheresh DA (2011) Tumor angiogenesis: molecular pathways and therapeutic targets. Nature medicine 17: 1359–1370. 10.1038/nm.2537 22064426

[6] Semela D, Dufour JF (2004) Angiogenesis and hepatocellular carcinoma. J Hepatol 41: 864–880. 15519663 10.1016/j.jhep.2004.09.006

[7] Tam K (2013) The Roles of Doxorubicin in Hepatocellular Carcinoma. ADMET & DMPK 1: 29–44.

[8] Folkman J (2006) Tumor suppression by p53 is mediated in part by the antiangiogenic activity of endostatin and tumstatin. Sci STKE DOI:10.1126/stke.3542006pe35. 10.1126/stke.3542006pe35 17003465

[9] Folkman J (2006) Antiangiogenesis in cancer therapy–endostatin and its mechanisms of action. Exp Cell Res 312: 594–607. 16376330 10.1016/j.yexcr.2005.11.015

[10] O'Reilly MS, Boehm T, Shing Y, Fukai N, Vasios G, et al (1997) Endostatin: an endogenous inhibitor of angiogenesis and tumor growth. Cell 88: 277–285. 9008168 10.1016/s0092-8674(00)81848-6

[11] Ling Y, Lu N, Gao Y, Chen Y, Wang S, et al (2009) Endostar induces apoptotic effects in HUVECs through activation of caspase-3 and decrease of Bcl-2. Anticancer Res 29: 411–417. 19331180

[12] Dong X, Zhao X, Xiao T, Tian H, Yun C (2011) Endostar, a recombined humanized endostatin, inhibits lymphangiogenesis and lymphatic metastasis of Lewis lung carcinoma xenograft in mice. Thorac Cardiovasc Surg 59: 133–136. 21480131 10.1055/s-0030-1250152

[13] Xu W, Ye P, Li Z, Shi J, Wang W, et al (2010) Endostar, a recently introduced recombinant human endostatin, inhibits proliferation and migration through regulating growth factors, adhesion factors and inflammatory mediators in choroid-retinal endothelial cells. Molecular Biology 44: 585–590. 20873226

[14] Folkman J (2006) Antiangiogenesis in cancer therapy–endostatin and its mechanisms of action. Experimental cell research 312: 594–607. 16376330 10.1016/j.yexcr.2005.11.015

[15] Cai W, Gambhir SS, Chen X (2008) Chapter 7. Molecular imaging of tumor vasculature. Methods Enzymol 445: 141–176. 19022059 10.1016/S0076-6879(08)03007-3

[16] Cai W, Chen X (2008) Multimodality molecular imaging of tumor angiogenesis. J Nucl Med 49 Suppl 2113S–128S. 18523069 10.2967/jnumed.107.045922

[17] Willmann JK, Van Bruggen N, Dinkelborg LM, Gambhir SS (2008) Molecular imaging in drug development. Nature Reviews Drug Discovery 7: 591–607. 18591980 10.1038/nrd2290

[18] Jaffer FA, Libby P, Weissleder R (2009) Optical and multimodality molecular imaging insights into atherosclerosis. Arteriosclerosis, thrombosis, and vascular biology 29: 1017–1024. 10.1161/ATVBAHA.108.165530 PMC2733228 19359659

[19] Baker M (2010) Whole-animal imaging: The whole picture. Nature 463: 977–980. 20164931 10.1038/463977a

[20] Tian J, Bai J, Bao S (2013) Optical Multi-Modality Molecular Imaging. Molecular Imaging: Springer. pp. 389–414.

[21] Bibby M (2004) Orthotopic models of cancer for preclinical drug evaluation: advantages and disadvantages. European Journal of Cancer 40: 852–857. 15120041 10.1016/j.ejca.2003.11.021

[22] Avella DM, Li G, Schell TD, Liu D, Zhang SS, et al (2012) Regression of established hepatocellular carcinoma is induced by chemoimmunotherapy in an orthotopic murine model. Hepatology 55: 141–152. 21898502 10.1002/hep.24652 PMC3243781

[23] Feng YX, Wang T, Deng YZ, Yang P, Li JJ, et al (2011) Sorafenib suppresses postsurgical recurrence and metastasis of hepatocellular carcinoma in an orthotopic mouse model. Hepatology 53: 483–492. 21274870 10.1002/hep.24075

[24] Blow N (2009) In vivo molecular imaging: the inside job. Nature Methods 6: 465–469.

[25] Ma X, Tian J, Yang X, Qin C, Zhu S, et al (2011) Research on liver tumor proliferation and angiogenesis based on multi-modality molecular imaging. Acta Biophys Sin 27: 355–364.

[26] Zhu S, Tian J, Yan G, Qin C, Feng J (2009) Cone beam micro-CT system for small animal imaging and performance evaluation. Journal of Biomedical Imaging DOI:10.1155/2009/960573. 10.1155/2009/960573 PMC2754077 19794829

[27] Tian J, Xue J, Dai Y, Chen J, Zheng J (2008) A novel software platform for medical image processing and analyzing. Information Technology in Biomedicine, IEEE Transactions on 12: 800–812. 10.1109/TITB.2008.926395 19000961

[28] Xue Z, Ma X, Zhang Q, Wu P, Yang X, et al (2013) Adaptive regularized method based on homotopy for sparse fluorescence tomography. Appl Opt 52: 2374–2384. 23670769 10.1364/AO.52.002374

[29] Hoffman RM (1999) Orthotopic metastatic mouse models for anticancer drug discovery and evaluation: a bridge to the clinic. Invest New Drugs 17: 343–359. 10759402 10.1023/a:1006326203858

[30] Killion JJ, Radinsky R, Fidler IJ (1998) Orthotopic models are necessary to predict therapy of transplantable tumors in mice. Cancer and Metastasis Reviews 17: 279–284. 10352881 10.1023/a:1006140513233

[31] Hu H, Liu J, Yao L, Yin J, Su N, et al. Real-time bioluminescence and tomographic imaging of gastric cancer in a novel orthotopic mouse model. Oncol Rep 27: 1937–1943. 10.3892/or.2012.1713 22407359

[32] Virostko J, Powers AC, Jansen ED (2007) Validation of luminescent source reconstruction using single-view spectrally resolved bioluminescence images. Appl Opt 46: 2540–2547. 17429468 10.1364/ao.46.002540

[33] Cheong SJ, Lee CM, Kim EM, Uhm TB, Jeong HJ, et al (2011) Evaluation of the therapeutic efficacy of a VEGFR2-blocking antibody using sodium-iodide symporter molecular imaging in a tumor xenograft model. Nucl Med Biol 38: 93–101. 21220132 10.1016/j.nucmedbio.2010.05.009

[34] Rehemtulla A, Stegman LD, Cardozo SJ, Gupta S, Hall DE, et al (2000) Rapid and quantitative assessment of cancer treatment response using in vivo bioluminescence imaging. Neoplasia (New York, NY) 2: 491. 10.1038/sj.neo.7900121 PMC1508085 11228541

[35] Xibo M, Zhaofei L, Xin Y, Qiujuan G, Shouping Z, et al (2011) Dual-modality Monitoring of Tumor Response to Cyclophosphamide Therapy in Mice with Bioluminescence Imaging and Small-animal Positron Emission Tomography. Molecular imaging 10: 278–283. 21501569 10.2310/7290.2010.00041

[36] Han D, Yang X, Liu K, Qin C, Zhang B, et al (2011) Efficient reconstruction method for L1 regularization in fluorescence molecular tomography. Appl Opt 49: 6930–6937. 10.1364/AO.49.006930 21173828

[37] Han D, Tian J, Zhu S, Feng J, Qin C, et al (2011) A fast reconstruction algorithm for fluorescence molecular tomography with sparsity regularization. Opt Express 18: 8630–8646. 10.1364/OE.18.008630 20588707

